# Fluid balance correlates with clinical course of multiple organ dysfunction syndrome and mortality in patients with septic shock

**DOI:** 10.1371/journal.pone.0225423

**Published:** 2019-12-02

**Authors:** Allen Chung-Cheng Huang, Tim Yu-Ting Lee, Meng-Cheng Ko, Chih-Hsien Huang, Tsai-Yu Wang, Ting-Yu Lin, Shu-Min Lin

**Affiliations:** 1 Department of Thoracic Medicine, Chang Gung Memorial Hospital, Chang Gung University, School of Medicine, Taipei, Taiwan; 2 Department of Internal Medicine, Chang Gung Memorial Hospital, Chang Gung University, School of Medicine, Taipei, Taiwan; 3 Department of Respiratory Therapy, Chang Gung Memorial Hospital, Chang Gung University, School of Medicine, Taipei, Taiwan; National Yang-Ming University, TAIWAN

## Abstract

**Introduction:**

Positive fluid balance is a prognostic factor for mortality in patients with sepsis; however, the association between cumulated fluid balance (CFB) and sepsis-induced multi-organ dysfunction syndrome (MODS) has yet to be elucidated. In this study, we sought to determine whether CFB is correlated with MODS and mortality in cases of septic shock.

**Methods:**

The study retrospectively recruited patients with septic shock from the intensive care unit of a tertiary care hospital. Multiple organ dysfunction syndrome (MODS) was identified as sequential organ failure assessment (SOFA) score ≥ 2 in more than one organ system. The CFB is the sum of all daily intake and output. An independent t-test, single and multivariate logistic regression, the receiver operating characteristic (ROC) curves, and the Pearson correlation coefficient were used to determine whether a relationship exists between CFB and the development of MODS and mortality.

**Results:**

Among the 104 patients enrolled in the study, 58 (55.8%) survived more than 28 days, and 73 (70.2%) developed MODS on day 3. The values of CFB in the first 24 hours and 72 hours after diagnosis of septic shock in patients with MODS were higher than these in patients without MODS (1086.6 ± 176.3 vs. 325.5 ± 205.7 ml, p = 0.013 and 2408 ± 361 vs. 873.1 ± 489 ml, p < 0.0001). In a multivariate logistic regression, the independent factors associated with the development of MODS on day 3 were APACHE II score at ICU admission (27.6 ± 7.6 in patients with MODS vs. 20.5 ± 6.4 in those without; O.R. 1.18; 95% C.1 I. 1.08–1.30; p < 0.001), disseminated intravascular coagulopathy (DIC) (n = 28; 38.4% vs. n = 2; 6.5%; O.R. 23.67; 95% C.I. 3.58–156.5; p = 0.001), and CFB in the first 72 hours (72-hr CFB) > median (1767.50ml) (n = 41; 56.2% vs. n = 11; 35.5%; O.R. 3.67; 95% C.I., 1.18–11.40; p = 0.024). Moreover, a multivariate logistic regression also identified neoplasm (n = 25; 54.3% vs. n = 17; 29.3%; O.R. 3.45; 95% C.I. 1.23–10.0; p = 0.019) and 72-hr CFB > median (n = 30; 65.2% vs. n = 21; 36.2%; O.R. 4.13; 95% C.I. 1.34–12.66; p = 0.013) as independent factors associated with 28-day mortality. 72-hr CFB values were strongly correlated with the SOFA score (r = 0.445, p < 0.0001). The area under the ROC curve revealed that 72-hr CFB has good discriminative power in associating the development of MODS (0.644, p = 0.002) and predicting subsequent 28-day mortality (0.704, p < 0.0001).

**Conclusions:**

72-hr CFB appears to be correlated with the likelihood of developing MODS and mortality in patients with septic shock. Thus, it appears that 72-hr CFB could perhaps be used as an indicator for MODS and a predictor for mortality in those patients.

## Introduction

Sepsis imposes a heavy healthcare burden and has a profound impact on survival outcomes worldwide [1]. Previous evidence has shown that early goal-directed therapies (EGDT) are associated with improved survival outcomes in patients with severe sepsis and septic shock [2]. EGDT involves aggressive fluid resuscitation, vasopressors, inotropes, and red-cell transfusion within 6 hours of diagnosis with the aim of achieving specific targets in terms of central venous pressure, mean arterial pressure, and central venous oxygen saturation. Since the publication of the above-mentioned study, numerous governing bodies have adopted strict treatment protocols for the management of septic shock [3, 4]. Fluid resuscitation in the initial stage of septic shock is crucial to the restoration of intravascular volume and the maintenance of hemodynamic stability. Nonetheless, the administration of fluids without appropriate monitoring can lead to fluid overload in patients with sepsis [5].

Emerging evidence suggests that persistent positive fluid balance is associated with adverse clinical outcomes and mortality in cases of sepsis [6–9]. The European SOAP study demonstrated that cumulative fluid balance (CFB) at 72 hours after the onset of septic shock is a strong predictor of survival [10]. Another recent study revealed that CFB at 72 hours after ICU admission (but not at 24 hours) was independently associated with an elevated risk of mortality [11]. In a study on sepsis patients with induced acute respiratory distress syndrome, it was found that adequate initial fluid resuscitation (defined as fluid administrated ≥ 20 mL/kg with central venous pressure ≥ 8mmHg before initiation of vasopressor therapy) and the conservative administration of fluid in later stages of treatment were associated with superior survival rates [9]. It therefore appears that fluid resuscitation in the initial management of sepsis should be timely and titrated according to the hemodynamic response. The aggressive and/or uncontrolled administration of fluids may be harmful to patients with sepsis.

Organ dysfunction is a hallmark clinical event of sepsis, and multiple organ dysfunction is the most severe outcome of the progression from simple infection to sepsis and septic shock. Sepsis-induced organ dysfunction often manifests as multiple organ dysfunction syndrome (MODS) with a high rate of mortality [12, 13]. A previous study reported that 78% of the patients with septic shock (in an intensive care setting) experienced organ system dysfunction; i.e., only 22% of the patients with severe sepsis presented single organ failure [14]. Previous reports have disclosed many important predictors for sepsis associated MODS including inflammatory cytokines, coagulatory abnormality, and endothelial injury [12, 15, 16]. These factors may cause alteration of microcirculation, decreased organ perfusion, and tissue hypoxia, which all lead to organ dysfunction [17]. Despite extensive research into the association between CFB and mortality in cases of sepsis, the association between CFB and sepsis-induced MODS has yet to be elucidated. In this study, we sought to determine whether CFB correlates with MODS and mortality in patients with septic shock. In addition, we initiated this study because fluid balance is readily accessible in everyday clinical practice in the ICU, and may be used as an early marker for MODS and mortality.

## Materials and methods

### Study population

**Fig 1:**
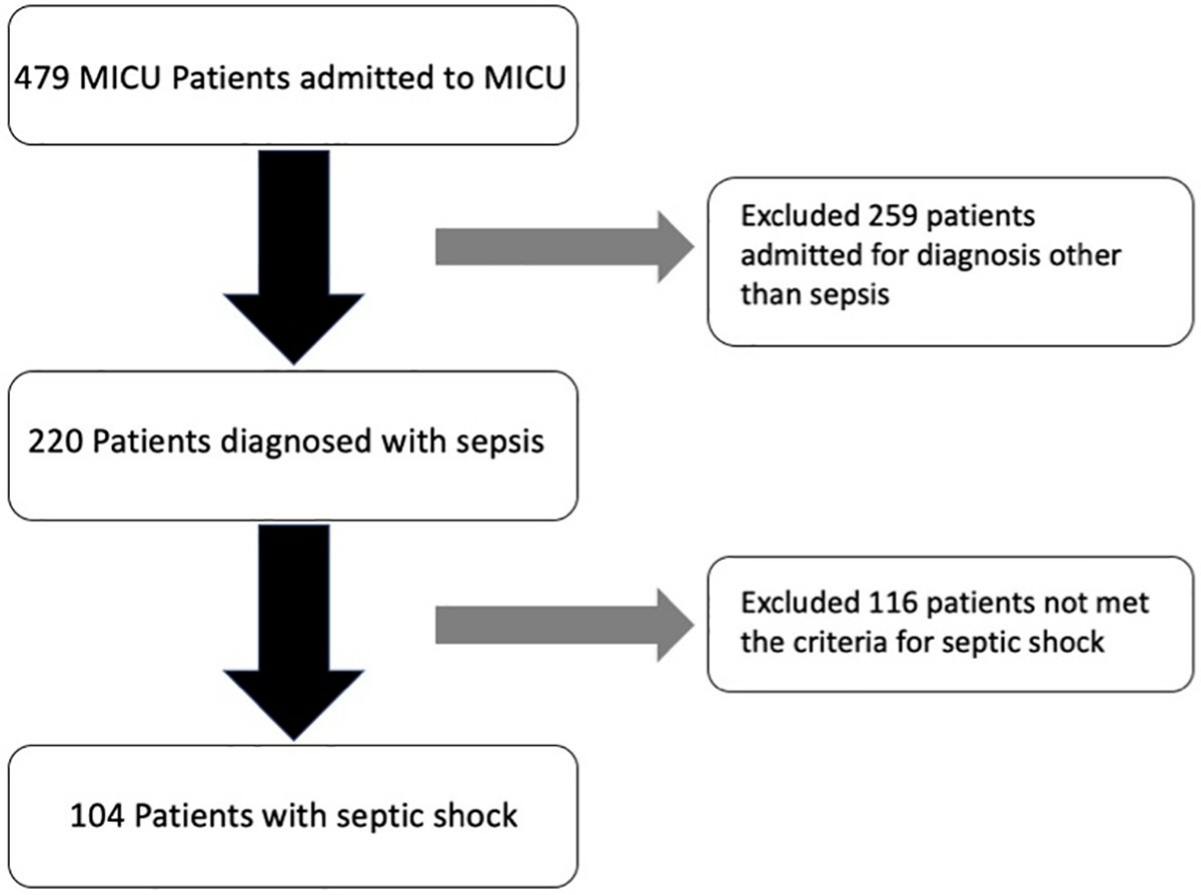
Patient flow chart.

Patient data were retrospectively collected from the 36-bed intensive care unit of a tertiary referral center in Taiwan (Chang Gung Memorial Hospital, Linkou Branch). The case-control study recruited 104 patients diagnosed with septic shock between January 1 and June 30, 2018 (Fig 1). All adult patients who met the following criteria were included in this study: (a) strongly suspected or proven infection supported by clinical evidence and/or positive bacteriological data followed by treatment with antibiotics; (b) clinically compatible with septic shock, as defined by acute organ dysfunction in the presence of an infection and the need for vasopressor treatment for more than 6 hours [18]. Patient baseline data included age, vital signs, blood gas analysis, organ failure count, and hematologic and biochemical tests. Acute Physiology and Chronic Health Evaluation II (APACHE II) scores and Sequential Organ Failure Assessment (SOFA) score [19] were used to assess illness severity. Underlying medical history was also collected, including diabetes mellitus, hypertension, neurologic disease, congestive heart failure, malignancy, and chronic airway obstruction disease.

### Definitions

Organ dysfunction was identified as an SOFA score ≥ 2 in one system in the presence of infection [18]. Multiple organ dysfunction syndrome (MODS) was identified as an SOFA score ≥ 2 in more than one system. The number of organ system failures was determined by SOFA scores on days 1 and 3 after the onset of septic shock. Persistent MODS was defined as cases with persistent MODS on days 1 and 3. No MODS was defined as cases of fewer than 2 organ failures on days 1 and 3. Resolved MODS was defined as cases of MODS on day 1 and fewer than 2 organ failures on day 3. Progressive MODS was defined as cases without MODS on day 1 and ≥2 organ failures on day 3. Bacteremia was defined as a positive yield of any bacteria in blood cultures during the ICU stay. The origin of sepsis was based on clinical diagnosis. Daily fluid intake was calculated as the sum of all intravenous and oral fluids. The daily fluid output was calculated as the sum of the volumes of urine output, ultrafiltration fluid, drain fluid, and estimated gastrointestinal losses. Insensible losses were also taken into account. The cumulative fluid balance is the sum of all input and output, accumulated over 24, 48, and 72 hours. The insensible loss was included in the sum of output. Disseminated intravascular coagulopathy (DIC) was diagnosed based on the modified International Society on Thrombosis and Haemostasis (ISTH) criteria, where platelet count, D-Dimer, fibrin degradation product and prothrombin time were collected. We also recorded the duration of the ICU stay, hospital stay, vasopressor use, ventilator use, and 28-day mortality. Vasopressor-free days and ventilator-free days were defined as the number of days the patient remained alive without the respective therapies [20]. Daily fluid balance was calculated by subtracting the total fluid output from the total intake. Day 1 was defined as the time between the point at which hypotension was first recorded and the following morning.

### Statistical analysis

All data were expressed as mean ± standard error of the mean (SEM) or percentage. Comparison of continuous and nominal variables between two groups was respectively performed using the Student t-test and the chi-square test. The receiver operating characteristic (ROC) curves, and Pearson correlation coefficient were used to determine whether a relationship exists between CFB and the development of MODS and mortality. One-way ANOVA was used for comparison of continuous variables among groups based on the SOFA score and the origin of organ failure. A p value of **<**0.05 using a two-sided test was considered statistically significant. To determine the predictors for MODS and 28-day mortality, univariate analysis was first performed. All variables with a p value **<**0.1 in univariate regression analysis were entered into a forward logistic regression analysis model to identify independent predictive factors for the development of MODS and 28-day mortality. All statistical analysis was performed using SPSS software, version 20.0 (SPSS, Inc., Chicago, IL).

### Ethics approval and consent to participate

The Chang Gung Medical Foundation Institutional Review Board (201900648B0) approved the study and waived the requirement for informed consent, due to the retrospective nature of the study.

### Funding

This project was supported by the Ministry of Science and Technology of Taiwan, R.O.C. (MOST 105-2314-B-182A-146-MY2) and research grants from Chang Gung Memorial Hospital, Taiwan (CIRPG3H0051)

## Results

### Demographic data

During the study period, there were 104 patients who met the inclusion criteria. Among them, 58 (55.8%) survived more than 28 days, as shown in Table 1. The mean age of the patients was 66.7±14.7 years, and most of them were male (67.3%). The mean APACHE II score of all patients at ICU admission was 25.4±7.9. The mean SOFA score was 10.2±3.1 on day 1 and 9.7±3.9 on day 3. MODS developed in 73 patients (70.2%) on day 3. Bacteremia was identified in 69 patients (66.3%). As for the origin of sepsis, pulmonary infection was identified in 72 (69.2%) patients, followed by primary bloodstream (25%), urinary tract (19.2%), soft tissue (13.5%), and intra-abdomen (6.7%).

**Table 1:** Characteristics and outcomes of patients with MODS and without MODS on day 3 following the onset of septic shock.

	All Patients n = 104	MODS(+) n = 73(70.2%)	MODS(-) n = 31(29.8%)	p value
**Patient characteristics**				
Age, year (mean)	66.7±14.7	68.0±13.5	63.7±17.0	0.17
Male, n (%)	70(67.3)	51(69.9)	19(61.3)	0.39
BMI (mean)	22.7±4.5	22.6±4.1	23.1±5.4	0.65
SOFA score on day 1 (mean)	10.2±3.1	11.0±2.8	8.3±3.0	0.00*
SOFA score on day 3 (mean)	9.7±3.9	10.9±3.4	6.8±3.4	0.00*
APACHE II score (mean)	25.4±7.9	27.6±7.6	20.5±6.4	0.00*
Bacteremia, n (%)	69(66.3)	49(67.1)	12(38.7)	0.79
DIC, n (%)	30(28.8)	28(38.4)	2(6.5)	0.001
72-hr CFB > median, n (%)	52(50.0)	41(56.2)	11(35.5)	0.005*
**Underlying conditions or risk factors**				
Diabetes mellitus, n (%)	28(26.9)	16(21.9)	12(38.7)	0.10
Hypertension, n (%)	47(45.2)	32(43.8)	15(38.4)	0.67
Smoking history, n (%)	18(17.3)	12(16.4)	6(19.4)	0.72
Chronic airway disease, n (%)	14(13.5)	11(15.1)	3(9.7)	0.46
Chronic kidney disease, n (%)	32(30.8)	25(34.2)	7(22.6)	0.22
Neoplasia, n (%)	42(40.4)	32(43.8)	10(32.3)	0.26
Immunosuppressive therapy, n (%)	10(9.6)	7(9.6)	3(9.7)	0.98
**Primary origin of sepsis**				
Pulmonary, n (%)	72(69.2)	54(74.0)	18(58.1)	0.13
Bloodstream, n (%)	25(24.0)	18(24.7)	7(22.6)	0.82
Musculocutaneous, soft tissue, n (%)	14(13.5)	9(12.3)	5(16.1)	0.60
Urinary tract, n (%)	20(19.2)	13(17.8)	7(22.6)	0.57
Intra-abdomen, n (%)	7(6.7)	4(5.5)	3(9.7)	0.43
**Clinical outcomes**				
ICU stay, days (mean)	15.6±14.0	15.9±14.0	14.9±14.4	0.73
Hospital stay, days (mean)	40.3±33.0	41.4±33.2	37.7±33.0	0.59
Ventilator duration, days (mean)	14.8 ±16.0	15.5±16.4	13.2±15.3	0.52
Ventilator-free in 28 days (mean)	12.2±11.8	10.7±11.7	15.8±11.5	0.04*
Vasopressor duration, days (mean)	5.34±8.7	5.3±5.1	5.2±14.0	0.97
Vasopressor-free in 28 days (mean)	18.1±11.6	16.2±12.1	22.4±8.9	0.01*
28-day mortality, n (%)	58(55.8)	38(52.1)	20(64.5)	0.42

*P-value <0.05 indicates statistical significance.

Abbreviations: APACHE II score: Acute Physiology and Chronic Health Evaluation II score; BMI: body mass index; MODS: multi-organ dysfunction syndrome; SOFA score: Sequential Organ Failure Assessment score; DIC: disseminated intravascular coagulopathy; ICU: intensive care unit. 72-hr CFB > median: cumulative fluid balance at 72-hour following the onset of septic shock > median (1767.50ml)

### Clinical outcomes

The mean duration of ICU stays and hospital stays were 15.6±14.0 days and 40.3±33.0 days, respectively. Patients who developed MODS had a similar mean ICU stay (15.9±14.0 vs. 14.9±14.4 days, p = 0.73) and hospital stay (41.4±33.2 vs. 37.7±33.0 days, p = 0.59), compared to those without MODS. The duration of ventilator use (15.5±16.4 vs. 13.2±15.3 days, p = 0.592 and vasopressor use (5.3±5.1 vs. 5.2±14.0 days, p = 0.97) were similar in the two groups. However, patients without MODS on day 3 functioned without these interventions for significantly longer durations: ventilator-free (10.7±11.7 vs. 15.8±11.5 days, p = 0.04) and vasopressor-free (16.2±12.1vs. 22.4±8.9 days, p = 0.01). Multivariate logistic regression analysis identified APACHE II score at MICU admission (OR, 1.18; 1.08–1.30, p<0.001, disseminated intravascular coagulopathy (DIC) (OR, 23.6; 3.58–156.5, p = 0.001) and 72hr-CFB > median (1767.50ml, OR, 3.67; 1.18–11.4, p = 0.024) as independent factors associated with the development of MODS (Table 2). After performing the same single and multivariate regression analysis, 72-hr CFB>median (1767.50ml, O.R., 4.13; 95%C.I., 1.34–12.66; p = 0.013) and neoplasm (O.R., 3.45; 95%C.I., 1.23–10.0; p = 0.019) were the only two independent predictors for 28-day mortality in patients with septic shock (Table 3).

**Table 2:** Univariate and multivariate analysis of risk factors associated with MODS in patients with septic shock.

Variables	Univariate O.R.	95% C.I.	P-value	Multivariate O.R.	95% C.I.	p-value
APACHE II score	1.15	1.07–1.23	<0.001	1.18	1.08–1.30	<0.001*
DM	0.44	0.17–1.10	0.10	0.32	0.09–1.09	0.068
DIC	9.02	1.99–40.78	0.001	23.67	3.58–156.5	0.001*
72-hr CFB>median	2.69	1.11–6.51	0.033	3.67	1.18–11.40	0.024*

*P-value <0.05 indicates statistical significance.

Abbreviations: CFB: cumulative fluid balance; C.I.: confidence interval; DM: diabetes mellitus; DIC: disseminated intravascular coagulopathy; MODS: multi-organ dysfunction syndrome; O.R.: odds ratio.

**Table 3:** Univariate and multivariate analysis of risk factors associated with 28-day mortality in patients with septic shock.

Variables	Univariate O.R.	95% C.I.	p-value	Multivariate O.R.	95% C.I.	p-value
Male gender	0.48	0.20–1.13	0.098	0.96	0.30–3.05	0.943
BMI	0.92	0.83–1.02	0.083	0.98	0.87–1.10	0.695
DM	4.07	1.49–11.17	0.007	3.06	0.90–10.48	0.075
Bacteremia	0.45	0.19–1.05	0.094	0.35	0.11–1.10	0.353
Neoplasm	1.63	1.09–2.46	0.015	3.45	1.23–10.0	0.019*
Day 1 SOFA score	1.21	1.05–1.40	0.005	1.11	0.93–1.33	0.266
APACHE II score	1.06	1.01–1.12	0.022	1.03	0.96–1.10	0.462
72-hr CFB>median	1.80	1.21–2.69	0.033	4.13	1.34–12.66	0.013*

*P-value <0.05 indicates statistical significance.

Abbreviations: APACHE II score: Acute Physiology and Chronic Health Evaluation II score; B.M.I. body mass index. CFB: cumulative fluid balance; C.I.: confidence interval; DM: diabetes mellitus; O.R.: odds ratio; BMI: body mass index; SOFA score: Sequential Organ Failure Assessment score

### Fluid balance

**Fig 2:**
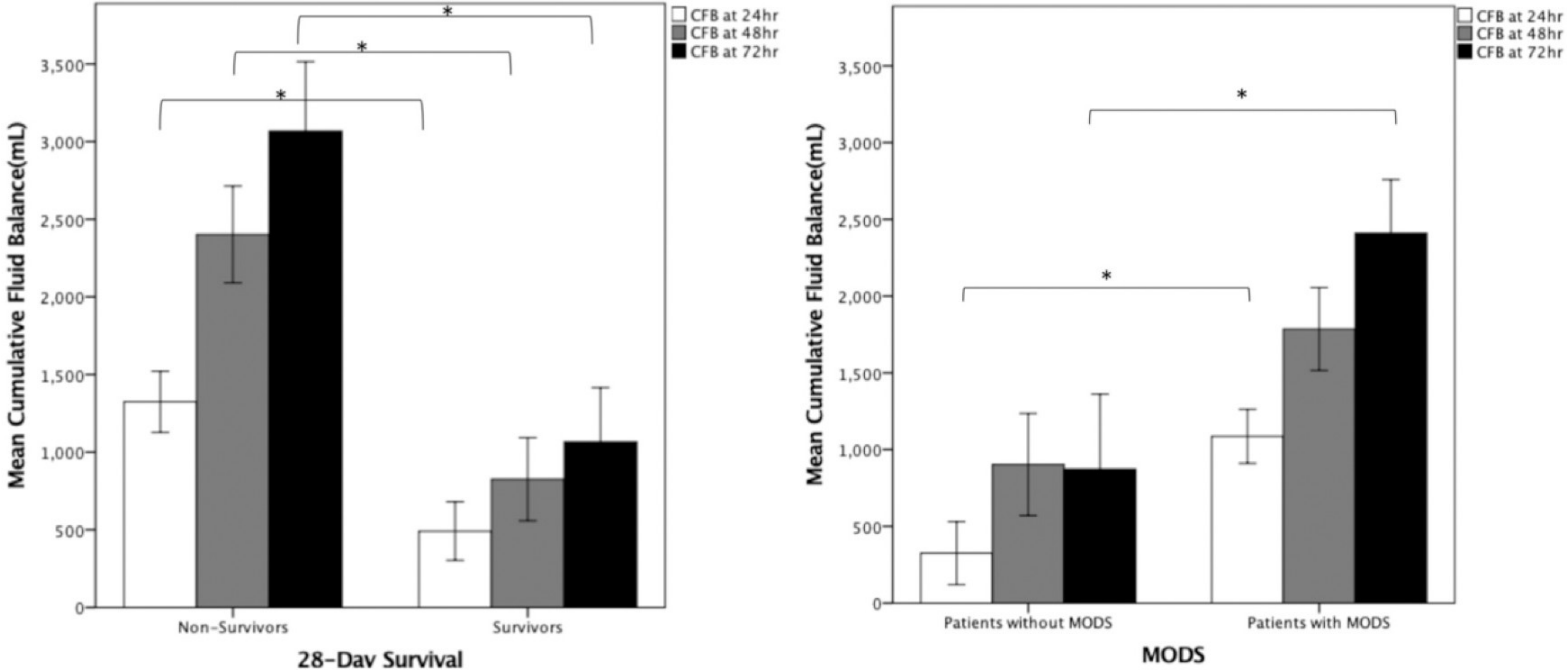
Relationship between cumulative fluid balance, MODS, and 28-day survival. **(A)** Cumulative fluid balances were significantly higher in non-survivors than in survivors of septic shock on 24 hr (open bar), 48 hr (gray bar), and 72 hr (black bar) (* indicates p<0.05); **(B)** Cumulative fluid balances were higher in patients with multi-organ dysfunction syndrome (MODS) than patients without MODS only on 24 hr (open bar) and 72 hr (black bar) (*p* = 0.035) but not on 48 hr (gray bar) of septic shock (* indicates p<0.05) (data expressed as mean ± SEM).

**Fig 3:**
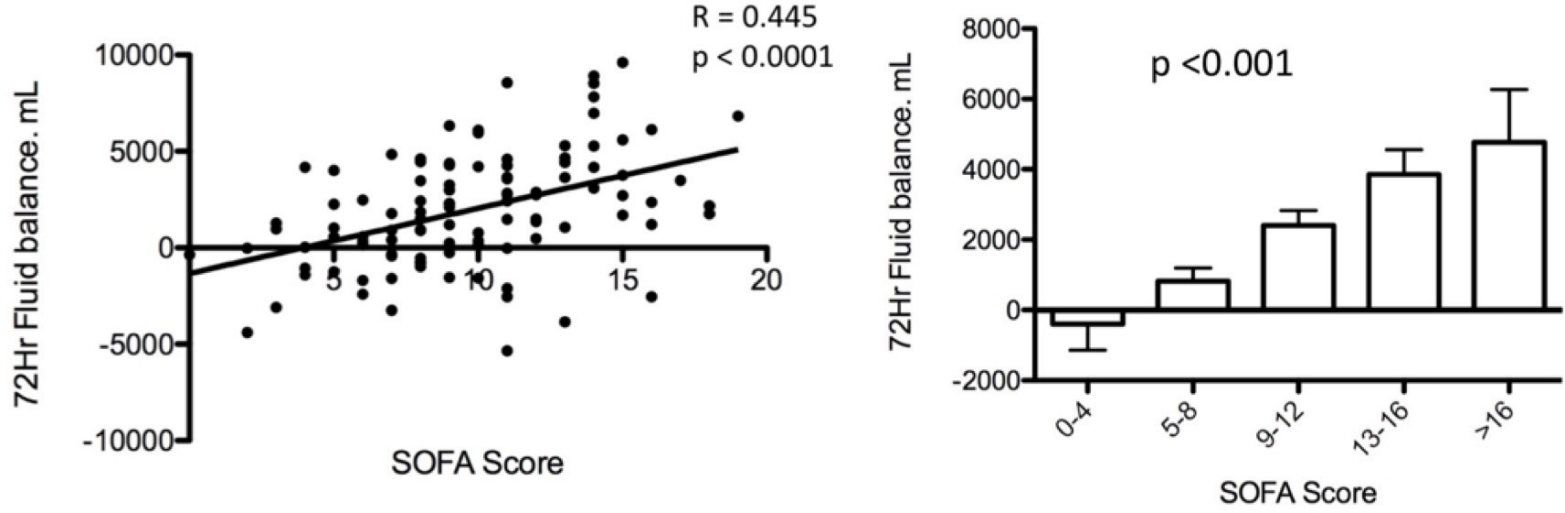
Relationship between 72-hr cumulative fluid balance (CFB) and SOFA score in patients with severe sepsis. **(A)** Amount of 72-hr CFB correlated well with SOFA score (r = 0.445, p < 0.0001) (data expressed as Pearson correlation); **(B)** The higher the SOFA score, the more 72-hr CFB was found (p<0.001 by ANOVA) (data expressed as mean ± SEM).

**Fig 4:**
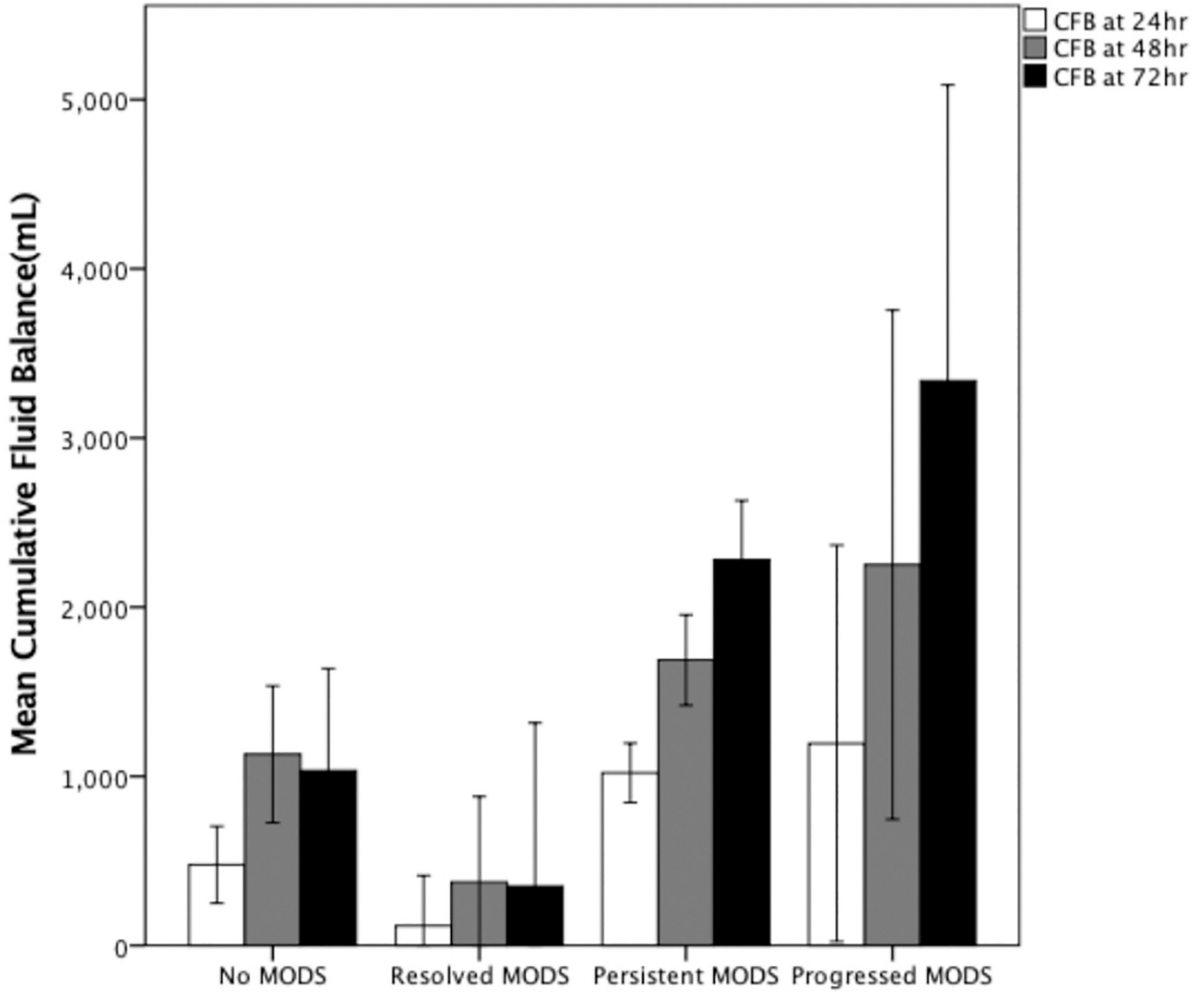
72-hr cumulative fluid balance (CFB) in patients with resolved persistent multiple organ dysfunction syndrome (MODS), progressive MODS, or without MODS from 24 hr to 72 hr after onset of septic shock. Open bars indicate 24-hr CFB; gray bar indicates 48-hr CFB; black bars indicate 72-hr CFB (data expressed as mean ± SEM).

**Fig 5:**
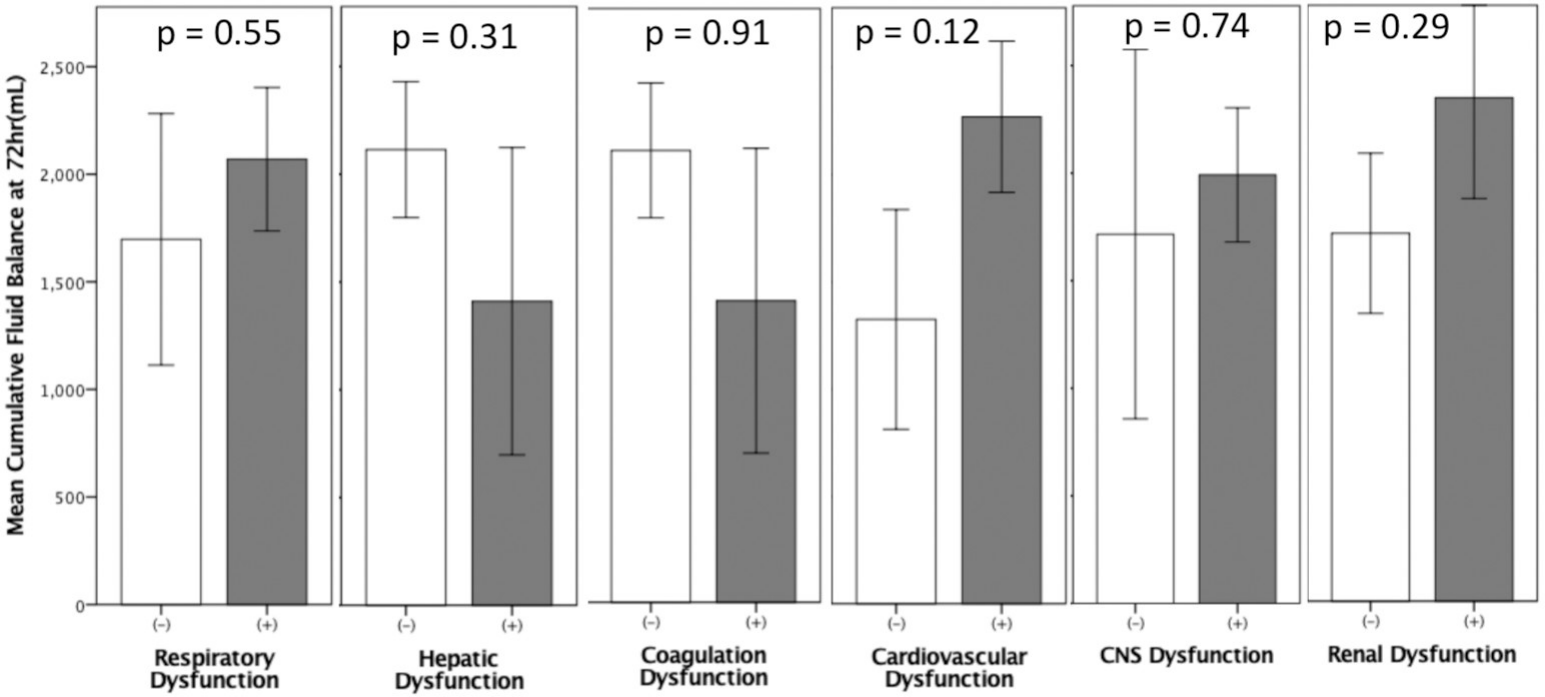
Comparison of 72-hr cumulative fluid balance (CFB) between patients with and without individual organ dysfunction. No significant difference in 72-hr CFB was observed between patients with and without individual organ failure (p values analyzed using Student t test; data expressed as mean ± SEM).

**Fig 6:**
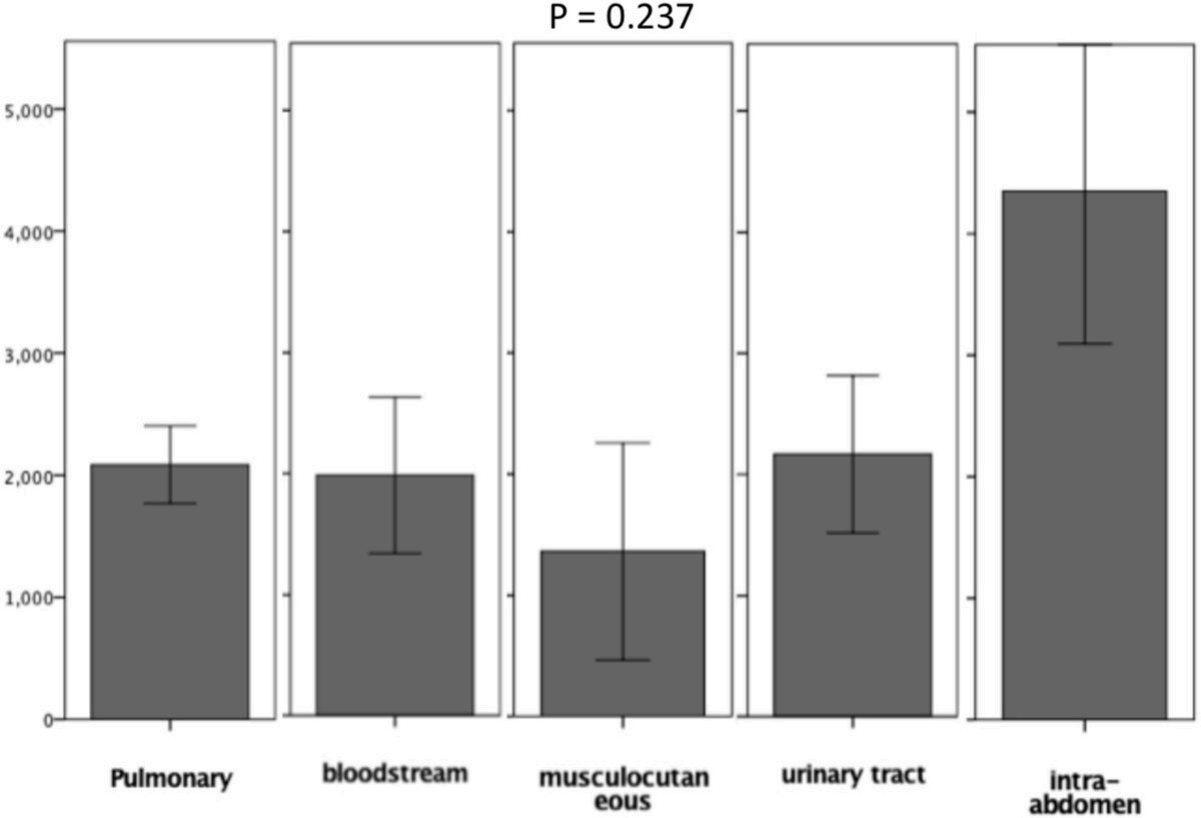
72-hr cumulative fluid balance (CFB) between patients with infections of various origin. Similar 72-hr CFB values were observed in patients with sepsis originating from intra-abdominal infection (4939.4±1256.7 ml), urinary tract infection (2167.4±648.2 ml), pulmonary infection (2086.3±317.3 ml), bloodstream infection (1986.8±645.1ml), and soft tissue infection (1360.9±895.8ml) (p = 0.273 using ANOVA).

CFB in non-survivors was significantly higher than in survivors: at 24 hours (1324±196.8 vs. 491.3±118.4 ml, p = 0.003), 48 hours (2401.8±311.6 vs. 825.7±267.9 ml, p = 0.0001), and 72 hours (3067.7±446.1 vs. 1066.7±348.8 ml, p = 0.0001). The cumulative output in non-survivors was also significantly lower at all three time points, as shown in Table 4 and Fig 2A. CFB in patients with MODS was significantly higher than in those without MODS: at 24 hours (1086.6±176.3 vs. 325.5±205.7, ml, p = 0.013) and 72 hours (2408±361 vs. 873.1±489 ml, p<0.0001) as shown in Fig 2B. In patients without MODS or resolved MODS, there was a drop in CFB between 48 and 72 hours. CFB at 72 hours correlated well with SOFA scores (r = 0.445, p<0.0001) (Fig 3A); i.e., a higher SOFA score was associated with a higher CFB at 72 hours (p<0.001 by ANOVA) (Fig 3B). In patients with persistent MODS or progressed MODS, there was an incremental increase in CFB at 24 hours, 48 hours, and 72 hours after the onset of septic shock (Fig 4). CFB was higher in patients with persistent MODS or progressed MODS than in patients without MODS or resolved MODS at 72 hours after the onset of septic shock (Fig 4). The area under the ROC curve revealed that 72hr-CFB has moderate discriminative power in associating the development of MODS (AUROC, 0.644,95% C.I., 0.530–0.758, p = 0.02, best cut-off value: 2272.0ml) and predicting mortality (AUROC, 0.704, 95%C.I., 0.602–0.806, p<0.001, best cut-off value: 347.5ml). We did not observe significant differences in 72-hr CFB between patients who did or did not present individual organ failure (Fig 5). The 72-hr CFB was similar among patients with septic shock due to intra-abdominal infection (4939.4±1256.7), urinary tract infection (2167.4±648.2ml), pulmonary infection (2086.3±317.3 ml), bloodstream (1986.8±645.1ml), and soft tissue infection (1360.9±895.8ml, p = 0.273 using ANOVA) (Fig 6).

**Table 4:** Comparison of cumulative fluid balance (i.e., input and output in first 72 hours following onset of septic shock).

	Survivors n = 58	Non-Survivors n = 46	p-value	MODS (-) n = 31	MODS (+) n = 73	p-value
**Fluid balance (mL), Mean** ±**SEM**						
First 24 hr	491.3 ± 118.4	1324 ± 196.8	0.003*	325.5 ± 205.7	1086.6 ± 176.3	0.013*
Cumulative 48 hr	825.7 ± 267.9	2401.8 ± 311.6	0.000*	903.2 ± 333.1	1785.9 ± 269.5	0.062
Cumulative 72 hr	1066.7 ± 348.8	3067.7 ± 446.1	0.001*	873.1 ± 489.3	2409.8 ± 349.7	0.016*
**Intake (mL), Mean ±SEM**						
First 24 hr	2139.6 ± 152.4	2395.4 ± 179.0	0.27	1990.9 ± 60.6	2363.9 ± 149.6	0.14
Cumulative 48 hr	4540.3 ± 225.8	5016.1 ± 280.2	0.18	4581.7 ± 306.7	4822.3 ± 217.3	0.53
Cumulative 72 hr	6968.8 ± 303.5	7454.4 ± 373.7	0.31	7018.9 ± 422.0	7235.5 ± 287.0	0.65
**Output (mL), Mean** ±**SEM**						
First 24 hr	1663.7 ± 171.1	1071.1 ± 94.6	0.003*	1665.3 ± 211	1289.2 ± 123.2	0.111
Cumulative 48 hr	3721.6 ± 239.2	2625.5 ± 174.2	0.000*	3681.7 ± 306.0	3047.9 ± 188.6	0.074
Cumulative 72 hr	5919.4 ± 311.5	4399.0 ± 275.9	0.000*	6152.2 ± 412.3	4862.5 ± 255.6	0.008*

*P-value <0.05 indicates statistical significance.

Abbreviations: MODS: multiple organ dysfunction syndrome

## Discussion

Our findings revealed changes in CFB with the evolution of MODS in patients with septic shock. 72-hr CFB remained unchanged in patients without MODS or with resolved MODS, whereas 72-hr CFB increased incrementally in patients with persistent MODS and progressive MODS. The CFB correlates well with the severity of organ dysfunction, as quantified by SOFA score. ROC curve analysis confirmed that 72-hr CFB has good discriminative power in predicting the development of MODS in patients with septic shock. Our results demonstrate a close association between CFB and MODS development/mortality in patients with septic shock.

Our observation of significantly higher CFB in non-survivors than in survivors is in agreement with findings in previous studies (5–8). We also observed significantly higher CFB in patients with MODS than in those without MODS at 24 hours and 72 hours after the onset of septic shock. However, there was no statistically significant difference between the 72-hr CFB in each individual organ dysfunction because most septic patients have more than just one dysfunctional organ system.

In the study, MODS on day 3 was not associated with mortality. In previous report [12], MODS on day 1 is associated with mortality in patients with septic shock. Similarly, our results demonstrated that organ dysfunction status on day 1 of septic shock, indicated by SOFA, is associated with mortality in patients with septic shock in univariate analysis. However, 72-hr cumulated fluid balance > median value and neoplasm were the only two independent predictors for mortality in multivariate analysis. Therefore, neoplasm rather than MODS on day 3 is the major contributor for mortality in the study population. In addition, the status of MODS from day 1 to day 3 may be affected by several factors including adequate antibiotic administration, presence of drug resistance microorganism, and management of septic shock. These factors may explain that MODS on day 3 was not associated with 28-day mortality.

Moreover, note that the association between CFB and MODS was largely overlooked in previous reports. There may be multiple mechanisms by which fluid overload contributes to organ dysfunction. First, elevated central venous pressure could decrease the pressure gradient in organs involved in systemic circulation, with the result that a decrease in blood flow could lead to organ dysfunction. Several studies have reported an association between venous congestion and acute kidney injury [21], liver dysfunction [22], and microcirculation failure [23]. Another possible mechanism is glycocalyx damage secondary to sepsis. The integrity of endothelidal glycocalyx plays a critical role in controlling fluid exchange through the endothelium [24]. Inflammation-mediated glycocalyx injury is responsible for changes in vascular permeability, resulting in the clinical manifestations of sepsis, including acute kidney injury, acute respiratory failure, and tissue edema [17, 25]. Fluid overload may also result in stretching of the heart, leading to the release of atrial and brain natriuretic peptides, which further exacerbate glycocalyx injury [26]. It is also possible that interstitial edema associated with fluid overload in individual organs could contribute to the development of compartment syndrome [27], which is closely linked to organ dysfunction, and particularly to acute kidney injury, which would further aggravate fluid imbalance.

The study revealed that DIC is an independent predictor of MODS in patients with septic shock. DIC is characterized by the widespread activation of coagulation, resulting in the intravascular formation of fibrin and ultimately thrombotic occlusion of vessels, followed by inconsistencies in oxygen supply and demand [28]. Previous evidence supports the assertion that persistent thrombotic activity in patients with DIC plays an important role in MODS and elevated mortality rates [12]. This is an indication of crosstalk between procoagulation and inflammatory mechanisms in the pathogenesis of organ failure and mortality in septic patients with DIC. MODS is a clinical entity characterized by generalized microvascular thrombosis. It may develop as an aspect of DIC syndrome, particularly in cases of gram-negative sepsis. A previous study on 1,789 ICU patients revealed a significant correlation between the SOFA score and the mortality of patients with DIC [29]. It has been shown that the activated and deregulated endothelium plays a pivotal role in the crosstalk between inflammation and coagulation in cases of sepsis [30]. It has also been reported that sepsis-induced endothelial damage with intravascular fluid re-distribution is associated with the development of septic shock [15].

Our results indicate that 72-hr CFB is associated with MODS and subsequent death in patients with septic shock. We found that CFB increased incrementally from day 1 to day 3 in patients with persistent MODS, whereas it remained stable in patients with resolved MODS. 72-hr CFB was also shown to have moderate discriminative power for predicting mortality. Overall, these findings suggest that 72-hr CFB may serve as an early predictor of survival in patients with septic shock. Serial measurements of CFB should make it possible to identify the patients who are in the greatest danger. An increase in CFB in the first 72 hours after the onset of septic shock would be indicative of persistent MODS (i.e., particularly vulnerable to mortality), whereas stable CFB would be an indication of resolved MODS. Our results require confirmation in the form of large-scale prospective studies; however, it appears that CFB measurements could perhaps be used as a marker by which to stratify patients in terms of severity. It is likely that the survival outcome of patients presenting a progressive increase in CFB could be improved through appropriate interventions. The interventions listed in Surviving Sepsis Guidelines [31] include the following: prompt analysis to confirm the source of infection, reassessment of antibiotic therapy using microbiological and clinical data, employing a low tidal volume and a limited inspiratory plateau pressure strategy for cases of acute lung injury (ALI)/acute respiratory distress syndrome (ARDS). Nonetheless, further studies will be required to confirm the applicability of CFB monitoring in the management of patients with severe sepsis.

The present study is limited by its retrospective design and relatively small sample size, which no doubt introduced biases in terms of patient selection and/or statistical analysis. We revealed a link between fluid overload and MODS and subsequent mortality; however, there is no way to deduce a cause-and-effect relationship between the two observations at this point. A prospective study comparing the effects of fluid restriction vs. liberal fluid administration will be required to determine whether fluid overload is directly harmful to organ function in patients with septic shock. In addition, central venous pressure (CVP), albumin, long-term hemodialysis and ARDS are important determining factor for CFB, but such information was not fully obtained in this study due to its retrospective nature.

In conclusion, we observed elevated 72-hr CFB values in cases of sepsis-induced MODS. CFB was shown to increase concomitantly with the progression of MODS in patients with septic shock, which suggests that fluid overload plays a critical role in progression from MODS to mortality. It appears that CFB (particularly 72-hr CFB) could be used to monitor the status of MODS in patients with septic shock as an early predictor of mortality.

## Supporting information

S1 File All patient data necessary to reproduce the results are provided. (XLSX)
